# Establishing 18F-PSMA-1007 Production in Armenia: Radiosynthesis and Comprehensive Quality Control

**DOI:** 10.5812/ijpr-169428

**Published:** 2026-03-18

**Authors:** Hayk Petrosyan, Marine Mnatsakanyan, Isabella Karapetyan, Andranik Manukyan, Gurgen Elbakyan, Davit Arshakyan, Nona Ginosyan, Armine Badeyan

**Affiliations:** 1 Radioisotope Production Center, 38/7 Halabyan str., 0036 Yerevan, Armenia; 2 Yerevan State University, 1 A. Manoogian str․, 0025 Yerevan, Armenia; 3 Alikhanyan National Science Laboratory, 2 Alikhanyan Brothers str., 0036, Yerevan, Armenia; 4 National Center of Oncology Named After V.A. Fanarjian, 76 Fanarjyan Str., 0052, Yerevan, Armenia

**Keywords:** 18F-PSMA-1007, PET, Nuclear Medicine, Radiopharmaceuticals, Prostate Tumor

## Abstract

**Background:**

18F-prostate-specific membrane antigen (18F-PSMA-1007) is a recognized positron emission tomography (PET) radiotracer commonly used for prostate tumor imaging due to its excellent tumor-to-background contrast and advantageous pharmacokinetics. Although 18F-PSMA-1007 has been extensively manufactured and used in many countries, it has not been available in Armenia until now. The aim of this work is to synthesize 18F-PSMA-1007 for the first time in Armenia for local clinical implementation and to demonstrate the reproducibility of the process.

**Objectives:**

Based on this, the objective of this work was to synthesize 18F-PSMA-1007 with high synthesis efficiency and chemical purity, which would enable us to perform precise studies in the field of prostate cancer diagnosis. The start of its production also represents a major advancement in the country's nuclear medicine capabilities.

**Methods:**

This study presents the initial report of the first successful production of 18F-PSMA-1007 in Armenia, detailing the synthesis method, quality control criteria, and adherence to good manufacturing practices (GMP) standards to ensure safe and efficient clinical use. An 18 MeV cyclotron was used to produce the 18F radioisotope needed for the production of the radiopharmaceutical. Starting ingredients, chromatographic resins, and a semi-automatic device were used in closed lead-shielded cells to synthesize 18F-PSMA-1007. The chemical reaction started after 18F ions were eluted into the reaction vessel using tetrabutylammonium bicarbonate (TBA). Residual solvents were then removed at 110°C for 5 minutes. The fluorination process was carried out using one-step radiolabeling at 95°C for 10 minutes, after which the resulting product was purified on a chromatographic resin for 10 minutes. Finally, 18F-PSMA-1007 was eluted into the dispensing cell. Comprehensive quality control analyses have been carried out in accordance with the guidelines of the European Pharmacopoeia (EP). To demonstrate the reproducibility of the synthesis of 18F-PSMA-1007 at the Radioisotope Production Center, as well as the quality control results, the work also presents the results of two additional syntheses and the corresponding quality control.

**Results:**

The successful synthesis of 18F-PSMA-1007 in Armenia, with a 52.2 % decay not corrected yield and 95 % purity, marks a significant advancement in the country’s nuclear medicine capabilities, providing highly sensitive and accurate PET examinations. The product met all specifications.

**Conclusions:**

The start of 18F-PSMA-1007 production locally has become a significant turning point for Armenian nuclear medicine. In addition to providing patients in the nation with access to cutting-edge PET diagnostic techniques, it also opens the door for the domestic radiopharmaceutical industry to grow and concentrate on exports.

## 1. Background

During the last decade, multiple radiolabeled molecules targeting the prostate-specific membrane antigen (PSMA) have been developed for both therapy and imaging applications (1, 2). Although only a limited number of 18F-labeled PSMA-targeted radiopharmaceuticals have been developed, the clinical application of those available has significantly enhanced the specificity for detecting prostate cancer and its treatment (3, 4). Prostate cancer, one of the leading causes of cancer-related mortality in men, represents the second most frequently diagnosed malignancy in the male population (5-7). Therapeutic strategies differ for localized, locally advanced, and metastatic disease; however, precise disease staging is critically dependent on imaging modalities with high sensitivity and specificity (8, 9). Emerging hybrid imaging techniques, particularly positron emission tomography (PET), are progressively replacing conventional abdominal imaging and planar scintigraphy for the detection and characterization of prostate cancer lesions (10-12).

18F-PSMA-1007 is a PET radiopharmaceutical with high sensitivity and specificity for the detection of both primary and metastatic prostate cancer lesions (13, 14). Compared to earlier PSMA-targeting ligands, it has several notable advantages, including minimal urinary excretion, which enhances lesion detectability in the pelvic region (15, 16). In contrast to 68Ga-PSMA11, 68Ga-PSMA-617, and the available fluorinated PSMA derivative, 18F-DCFPyL, 18F-PSMA-1007 has the distinct advantage of not being eliminated by the kidneys and having low activity in urine. This may help clinicians make decisions in cases of local recurrence or obscure lesions close to the ureter or bladder (17). The body distributes 18F-PSMA-1007 at a similar rate to 68Ga-PSMA-617 because of their similar structures. This enables the use of 18F-PSMA-1007 to assess a patient's suitability for 177Lu-PSMA-617 therapy. Another advantage is that 18F-PSMA-1007 radiopharmaceuticals make it possible to carry out large-scale production, which allows for more patient studies compared to the limited amount available from generator-produced 68Ga (18). Also, the longer half-life of the 18F radioisotope, which is about 109 minutes, makes it possible to produce the isotope in a central location and then send it to other medical centers for use (19). Clinical experience and international studies have demonstrated that the use of 18F-PSMA-1007 significantly improves the management of prostate cancer by enabling more accurate assessment of metastatic disease and facilitating optimized planning of targeted therapeutic interventions (20-22).

18F-PSMA-1007 binds to PSMA receptors expressed throughout the body and located predominantly on the surface of prostate cancer cells (23, 24). The transmembrane glycoprotein is the extracellular portion of PSMA that is exposed to circulating radiotracers (25). The radiotracer accumulates after binding of 18F-PSMA-1007 to this portion with high specificity and subsequent intracellular entry (26, 27). This mechanism allows detection of even small metastatic foci and provides a high signal-to-background ratio (28, 29). The ability to chemically bind to PSMA is due to the low molecular weight of the 18F-PSMA-1007 with a Glu-Urea-Lys structure (30). In addition, the molecule has a lipophilic side chain, which causes its minimal excretion in the urine (31). The 18F radioisotope is attached to a prosthetic group, which is usually part of a fluorinated or fluorobenzyl moiety. This structural combination ensures high stability in vivo, strong binding to the target, and biodistribution (32, 33).

In the world's leading centers for the production of radiopharmaceuticals, 18F-PSMA-1007 is synthesized using automatic or semi-automatic modules, which ensures radio-safety, as well as compliance with full GMP requirements (34, 35). The most popular modules are the GE FASTLAB, IBA Synthera® and Trasis AllInOne®, which allow achievement of high radiochemical yields and purity of 18F-PSMA-1007 with minimal operator intervention (15, 36, 37).

Apart from the fact that the introduction of the production and use of 18F-PSMA-1007 in Armenia is a significant achievement for the country's healthcare system, it also has strategic significance, as it reduces dependence on foreign centers and makes it possible to conduct very targeted research on prostate cancer and its metastatic lesions domestically. This is a significant step in the direction of providing nuclear medicine services in the Republic of Armenia, with domestic production of radiopharmaceuticals and exportation of those to the region's neighboring countries.

## 2. Methods

The 18F radioisotope required for the synthesis of the 18F-PSMA-1007 radiopharmaceuticals was obtained using an 18 MeV cyclotron [Cyclone 18Twin (38, 39), IBA, Belgium] by proton irradiation of 3.5 mL of enriched water (H2O18, CMR, Russia) with the nuclear reaction 18O(p,n)18F during 30 minutes. The synthesis of 18F-PSMA-1007 was carried out in closed lead-shielded cells - Hot Cell (Comecer, Italy) using a Synthera V2 (IBA, Belgium) semi-automatic device, and the starting materials and chromatographic resins were placed on the Integrated Fluidic Processor (IFP, ABX, Germany) designed for the synthesis.

### 2.1. 18F-PSMA-1007 synthesis

**Figure 1.**
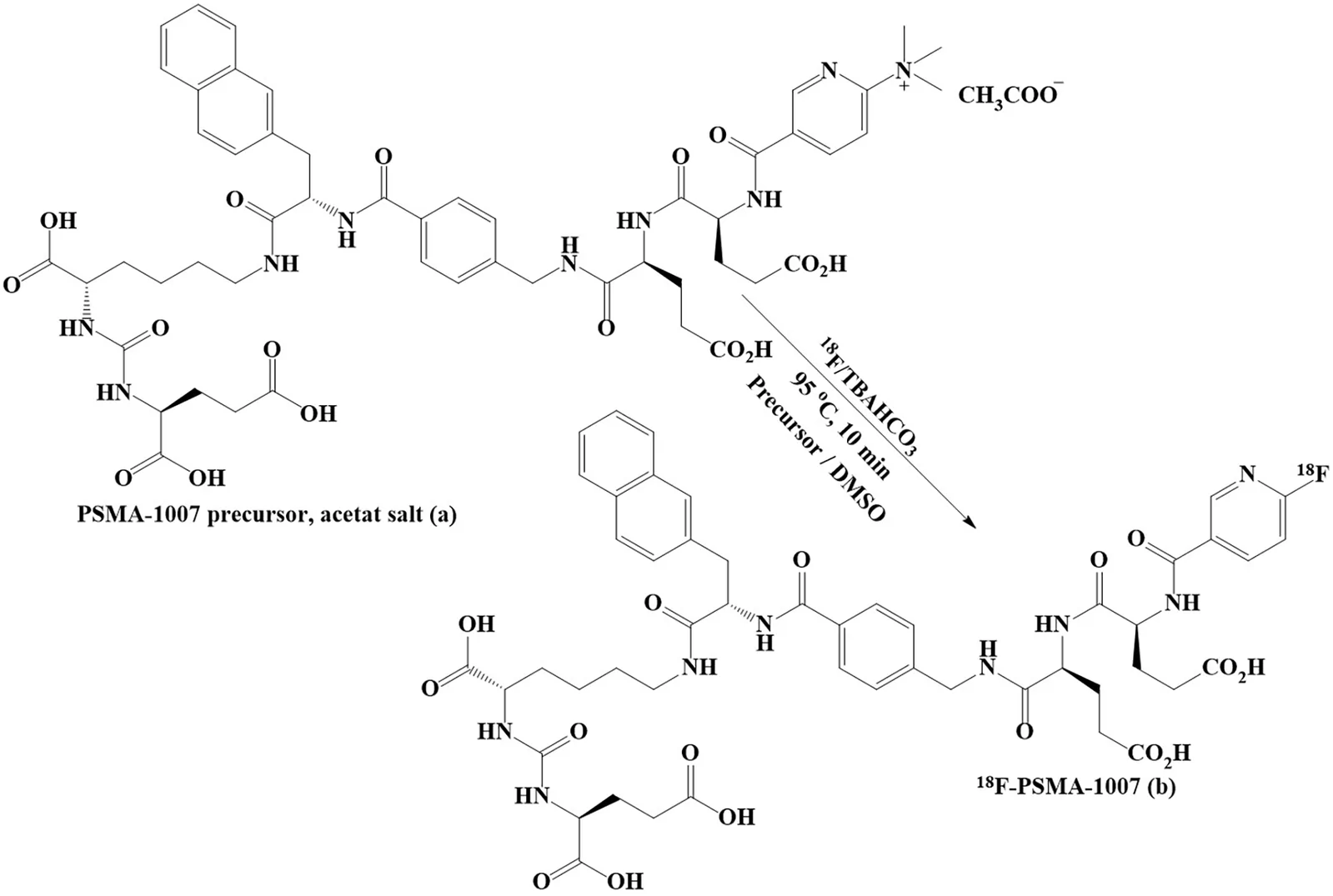
Pathway for nucleophilic fluorination of the acetate salt of the PSMA-1007 precursor (A) to form ^18^F-prostate-specific membrane antigen (^18^F-PSMA-1007) (B).

After trapping of 18F ions on anion exchange resin containing quaternary ammonium functional groups (QMA, Waters, USA), the elution of radioactive ions into the reaction vial was carried out with a 0.6 mL aqueous solution of tetrabutylammonium bicarbonate (ABX, Germany). Evaporation of the solvents at 110°C and under nitrogen flow was carried out, which took 10 minutes. The subsequent nucleophilic fluorination (Figure 1) process was carried out by adding 1 mg of the PSMA acetate salt (precursor, ABX, Germany) dissolved in 1.5 mL of dimethyl sulfoxide (DMSO, ABX, Germany). The fluorination process lasted for 10 minutes.

**Figure 2.**
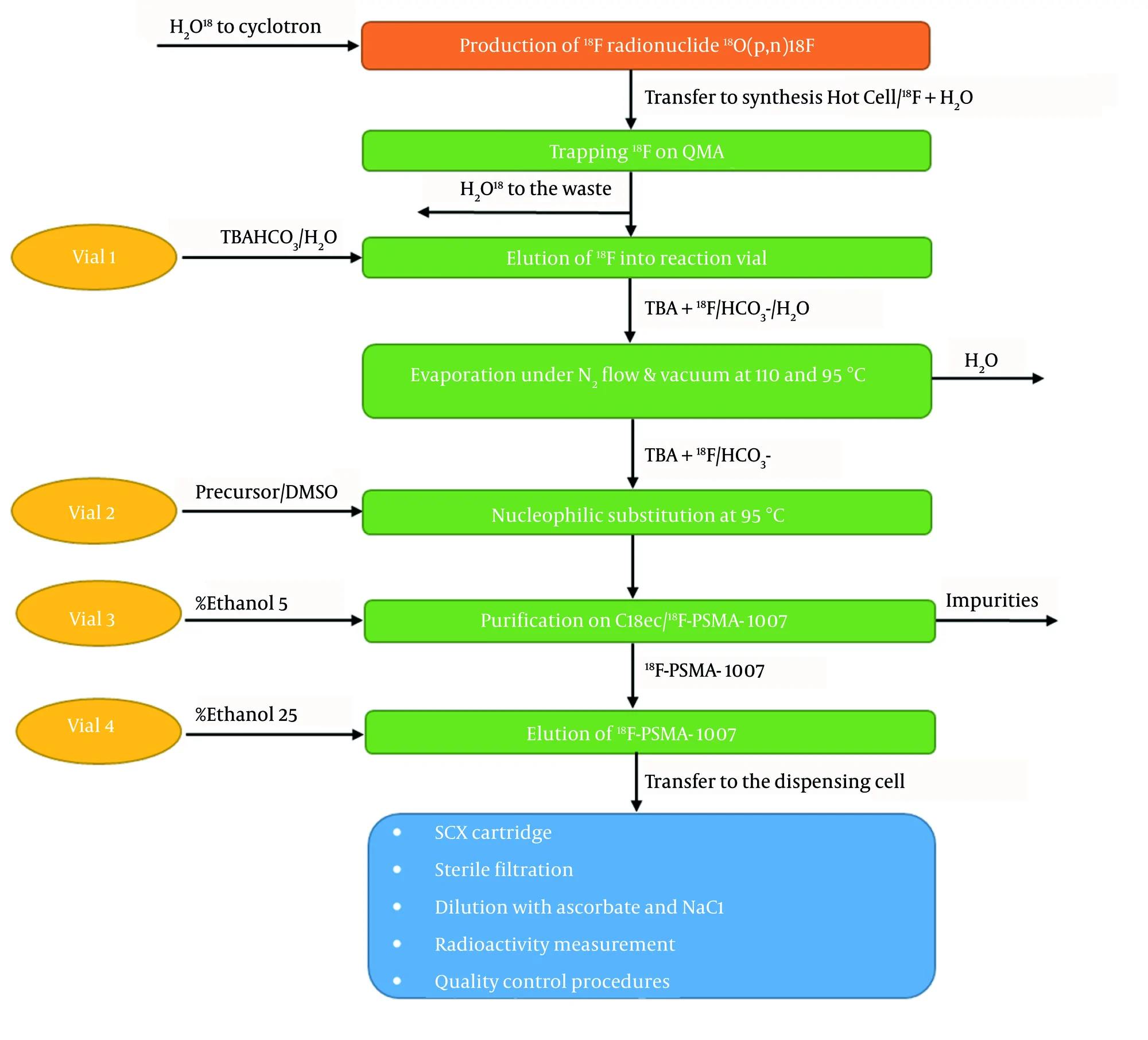
Flow chart for ^18^F-prostate-specific membrane antigen (^18^F-PSMA-1007) synthesis procedures

After that, the solution was diluted with a 6 mL 5.5 % ethanol solution and sent to a C18ec reversed-phase chromatographic resin (ABX, Germany) under a nitrogen flow. Subsequently, the sorbed radiopharmaceutical was purified with a 6 mL 5.5 % ethanol solution (ABX, Germany) four times. The final elution of the 18F-PSMA-1007 radiopharmaceutical from the C18 resin was performed with a 6 mL 25 % ethanol solution (ABX, Germany), and then transferred to a dispensing cell (Comecer, Italy) and subsequent sterilization. Before sterilization, the solution passed through SCX (strong cation exchange) resin. To prevent radiolysis and subsequent formation of free 18F ions, the final solution was diluted with a pre-added sodium ascorbate solution (15 mL). All reagents used in the synthesis were obtained from the manufacturer in a sterile state. The entire production process of the radiopharmaceutical 18F-PSMA-1007 is shown in Figure 2. 

### 2.2. Quality Control

The quality control process was carried out in accordance with the requirements of the European Pharmacopoeia, using appropriate physicochemical and biological methods. The appearance and pH of the radiopharmaceutical were determined by visual inspection and pH meter 765 Calimatic (Knick, Germany) equipment, respectively. Thin layer chromatography (TLC) was used to determine residual TBA levels, and the tetrabutylammonium hydroxide 30-hydrate (Thermo Fisher, United States) was used as a reference standard. For radionuclide identification, the isotope energy and half-life were measured using a multichannel analyzer (MUCHA, Elysia-Raytest, Germany) and the ISOMED 2010 (NUVIA Instruments, Germany) equipment, respectively. An Agilent 6850 gas chromatograph was used for the quantitative determination of residual solvents in accordance with International Council for Harmonisation (ICH) Q3C guidelines and EP requirements. Radiochemical purity was assessed using Mini Gita (Elysia-Raytest, Germany) radio-TLC equipment. The quantitative presence of bacterial endotoxins in the radiopharmaceutical was determined using the Endosafe PTS (Charles River Laboratories, USA) device, and the sterility test was performed using the EP Sterility Standard Test method. The sterility test was performed in a sterility laboratory, and a laminar airflow cabinet (A grade) was used to prevent accidental contamination during the procedure. These conditions are identical to those required for the aseptic production of pharmaceutical products. The sterility test was performed after the disappearance of radioactivity, which is permitted by the EP.

## 3. Results

### 3.1. 18F-PSMA-1007 Synthesis

**Figure 3.**
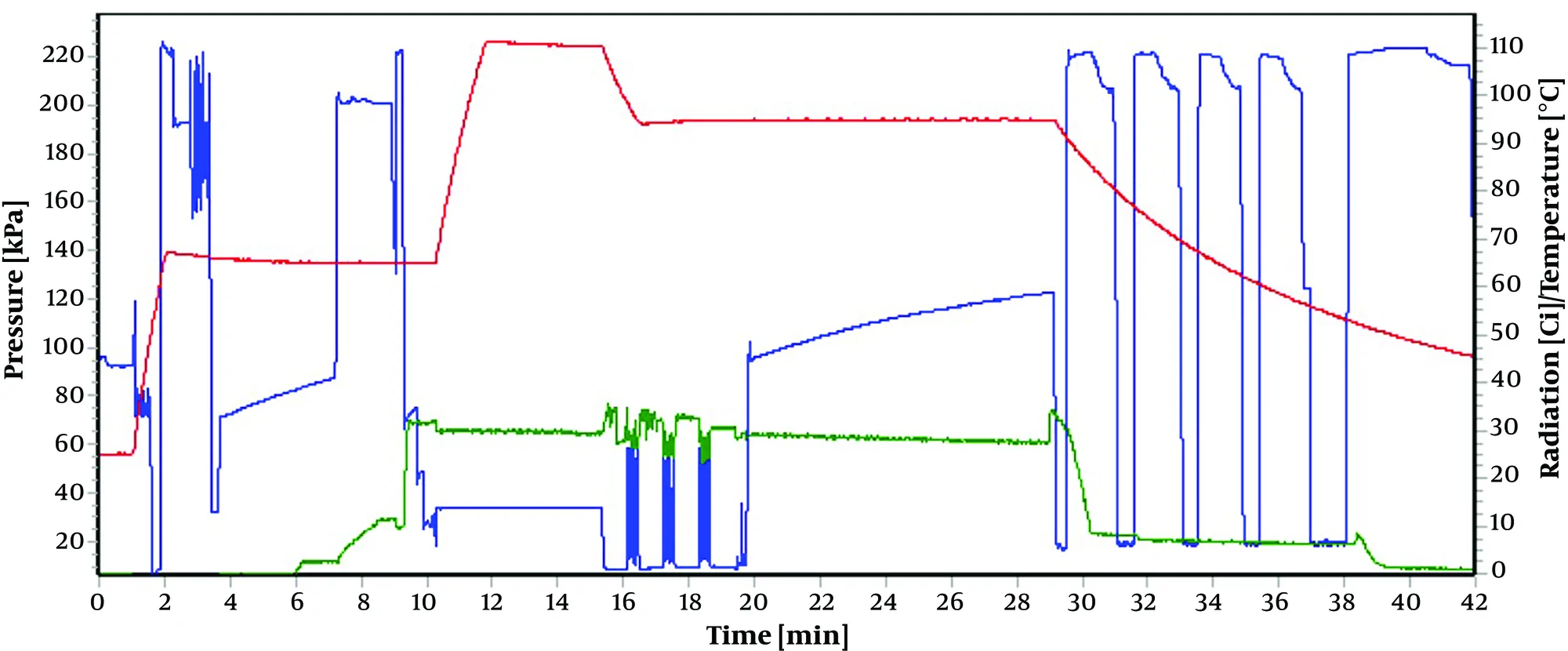
^18^F-prostate-specific membrane antigen (^18^F-PSMA-1007) synthesis report by Synthera [V2: Red-temperature, blue-pressure, green-radioactivity (10 times actual scale, in Curie-Ci)]

After 30 minutes of proton irradiation of the enriched water, 101 gigabecquerel (GBq) of radioactivity was generated as a result of the 18O(p,n)18F nuclear reaction. A volume of 3.5 mL was transferred via ultrapure helium flow to a V-Vial located in the Hot Cell of the production room, where a CRC-55tPET dose calibrator (Capintec Inc., USA) recorded 95 GBq of 18F radioactivity. Chemical synthesis started with 90 GBq of radioactivity and lasted 30-35 minutes. After transferring the obtained 18F-PSMA-1007 to a dispensing cell with high-purity nitrogen flow, 47 GBq radioactivity of radiopharmaceutical was recorded by VDC-505 dose calibrator (Comecer, Italy). The results obtained show that the decay not corrected radiochemical yield of 18F-PSMA-1007 (starting from the chemical synthesis itself) was 52.2 %. Figure 3 shows the process conditions (temperature, pressure, and radioactivity) during the chemical synthesis of 18F-PSMA-1007 using the Synthera V2 equipment. It is also important to note that the yields of the two syntheses performed to prove the reproducibility of the process were 56.69 % and 51.4 %, respectively.

**Figure 4.**
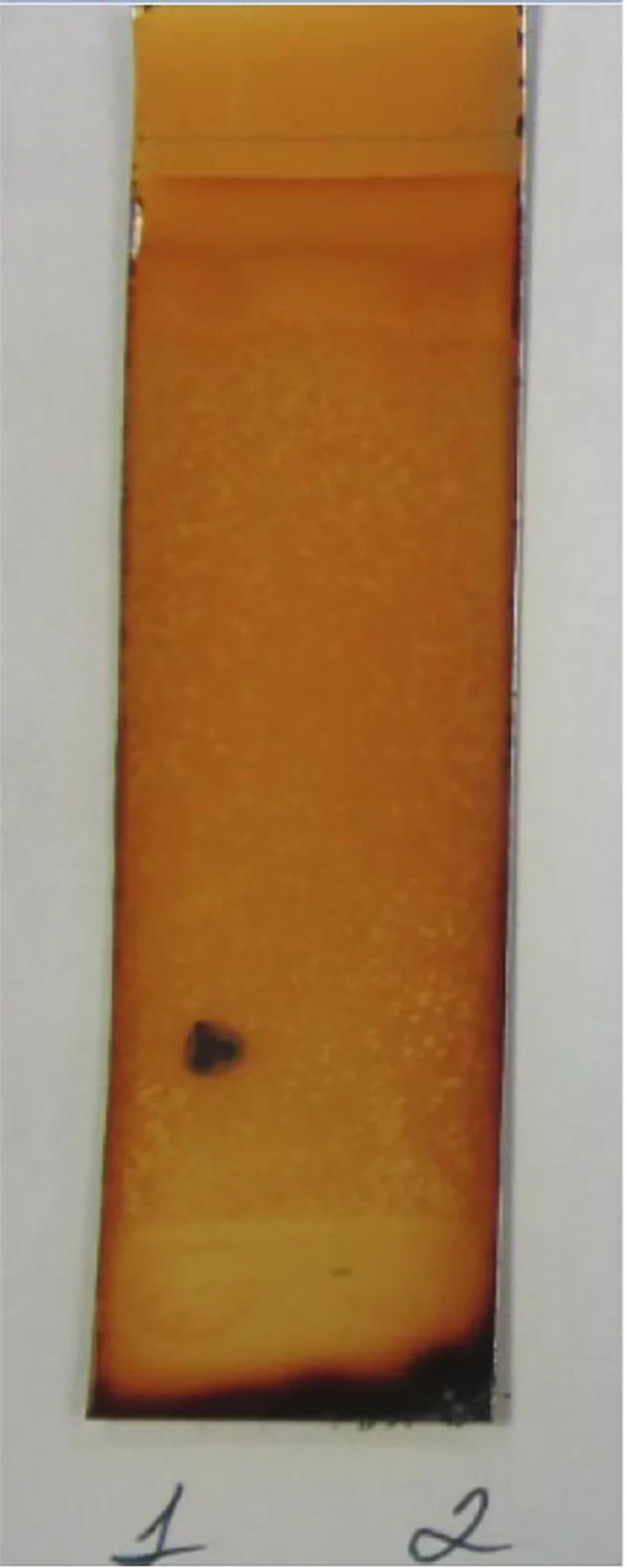
Thin layer chromatography (TLC) of tetrabutylammonium bicarbonate (TBA) for reference (1) and ^18^F-prostate-specific membrane antigen (^18^F-PSMA) solutions (2).

External examination (appearance) of the resulting radiopharmaceutical solution confirmed that it was colorless, and the pH value was 6.42, which falls within the permissible range for intravenous quality solution. There were no TBA spots found on the sample part of TLC “Silica gel 60” paper (Merck, Germany) (Figure 4), according to the chromatographic image developed using a methanol-ammonia (9:1) mobile phase and visualized using iodine vapor. It should be mentioned that the reference solution had a retardation factor (Rf) of 0.1-0.2 and contained 0.22 mg/mL of tetrabutylammonium ions. It is important to remember that the TBA content is evaluated qualitatively by comparing the color intensity of the sample spot to that of the reference.

**Figure 5.**
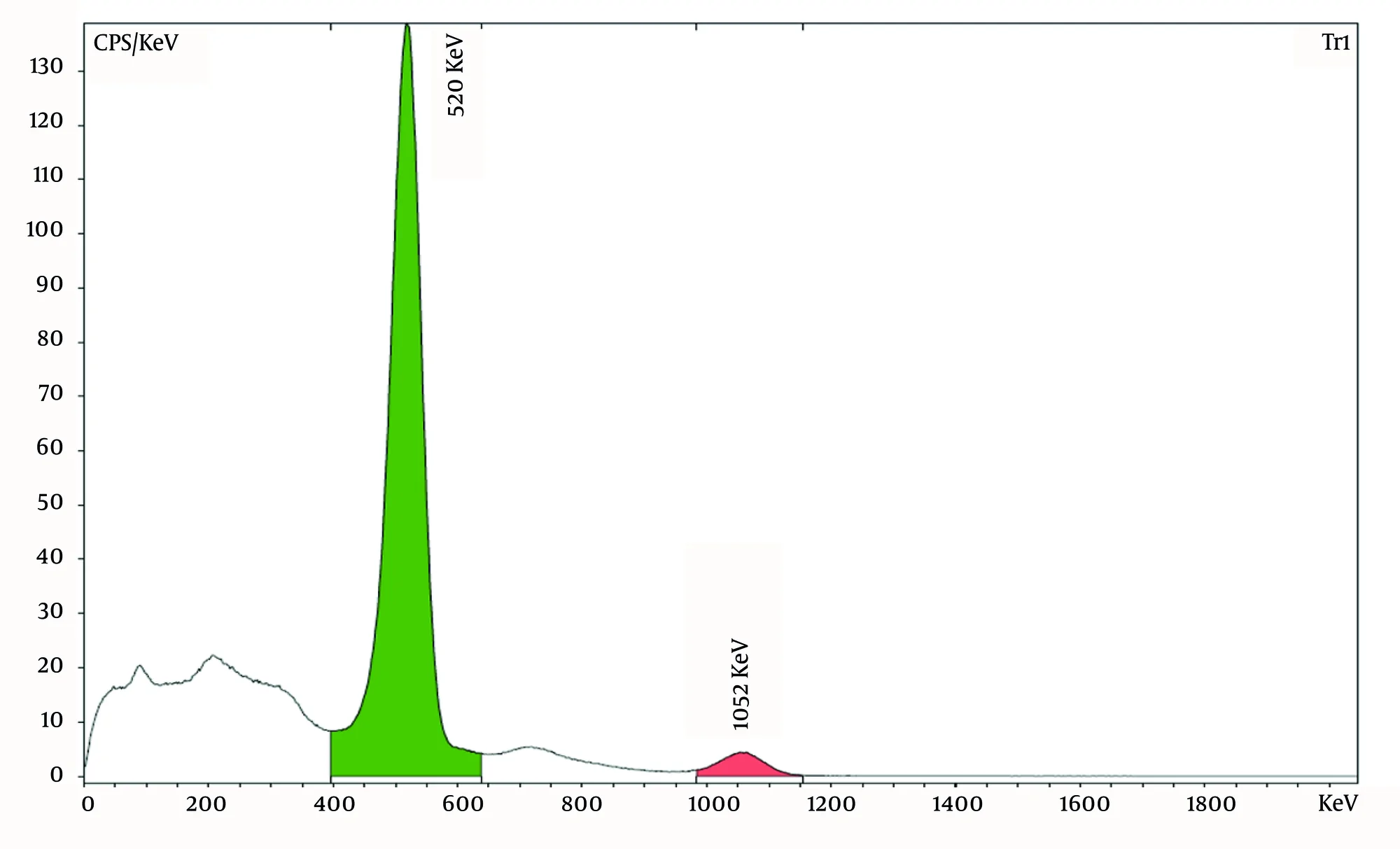
γ spectrum of ^18^F recorded by multichannel analyzer (MUCHA)

Measurements performed by γ-spectrometry confirmed the presence of the 18F isotope and radionuclidic purity. According to these measurements, the photon energy was 520 keV, the sum of the peaks energies was 1052 keV (Figure 5). Measurements performed using the ISOMED 2010 ionization chamber system confirmed a half-life of 107.4 minutes for 18F.

**Figure 6.**
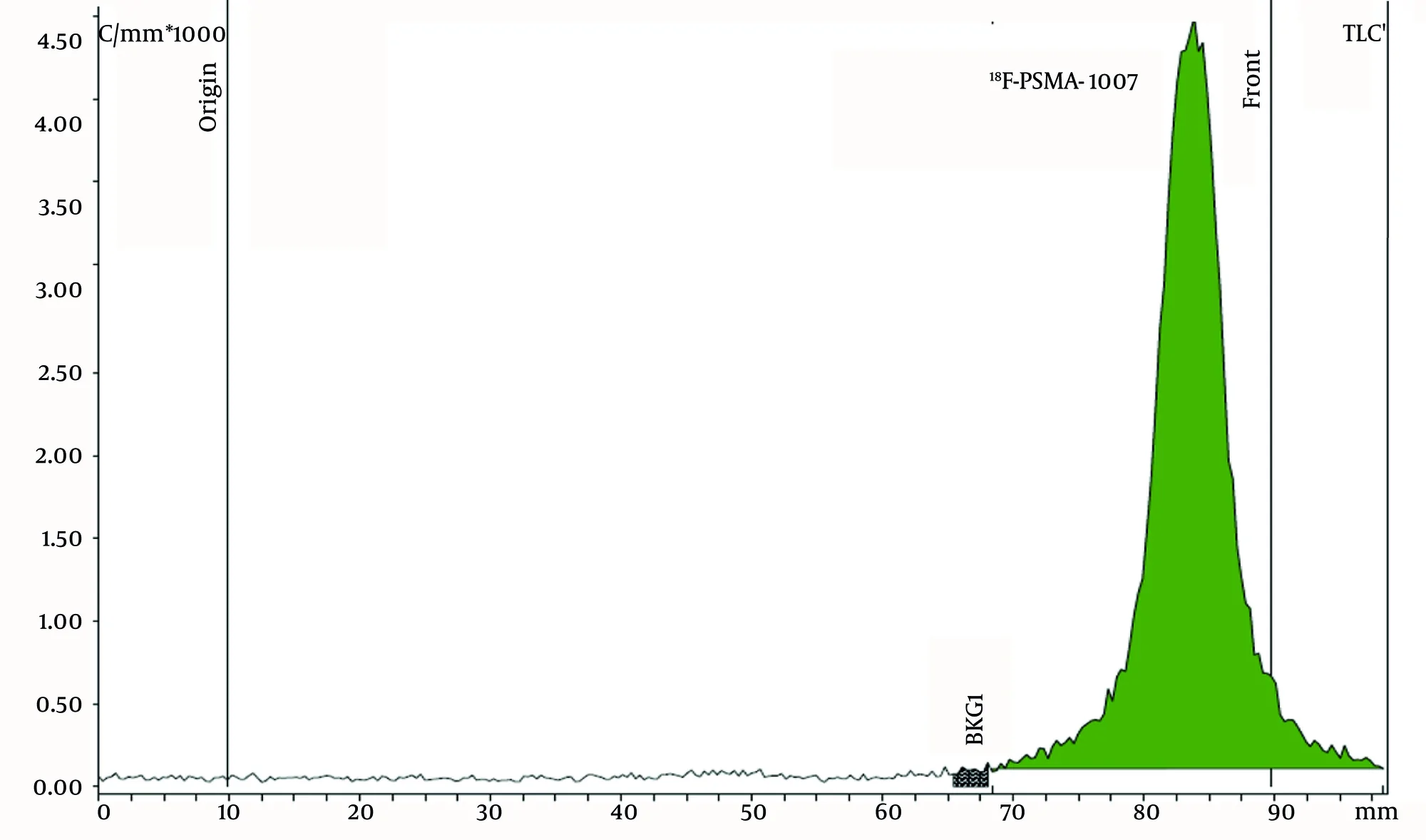
Radio-thin layer chromatography (TLC) chromatogram of ^18^F-prostate-specific membrane antigen (^18^F-PSMA-1007) obtained with MiniGita

As mentioned above, a Radio-TLC instrument (MiniGita) was used for radiochemical purity assessment. After developing the TLC paper with a mobile phase consisting of acetonitrile and water (60:40, v/v), which took approximately 20 minutes, the paper was placed in the center of the Radio-TLC detector for measurement, for a 2-minute measurement. The resulting radio chromatogram showed 95 % of the radioactivity corresponded to the target compound 18F-PSMA-1007 (Figure 6). 

The Rf was 0.926, which is consistent with the reference standard. It should be noted that the only potential radiochemical impurity compound after the synthesis of this radiopharmaceutical in question is free ions 18F, the content of which, according to the requirements of the European Pharmacopoeia, should not exceed 9 %. In the present case, no free 18F was detected on the radio chromatogram, which confirms the high radiochemical purity of the product. This also demonstrates the effectiveness of the buffer used - sodium ascorbate, which prevents the phenomenon of radiolysis and prevents the formation of free ions 18F in the radiopharmaceutical solution, which are capable of detecting “fake” accumulations in the body during PET examination.

**Figure 7.**
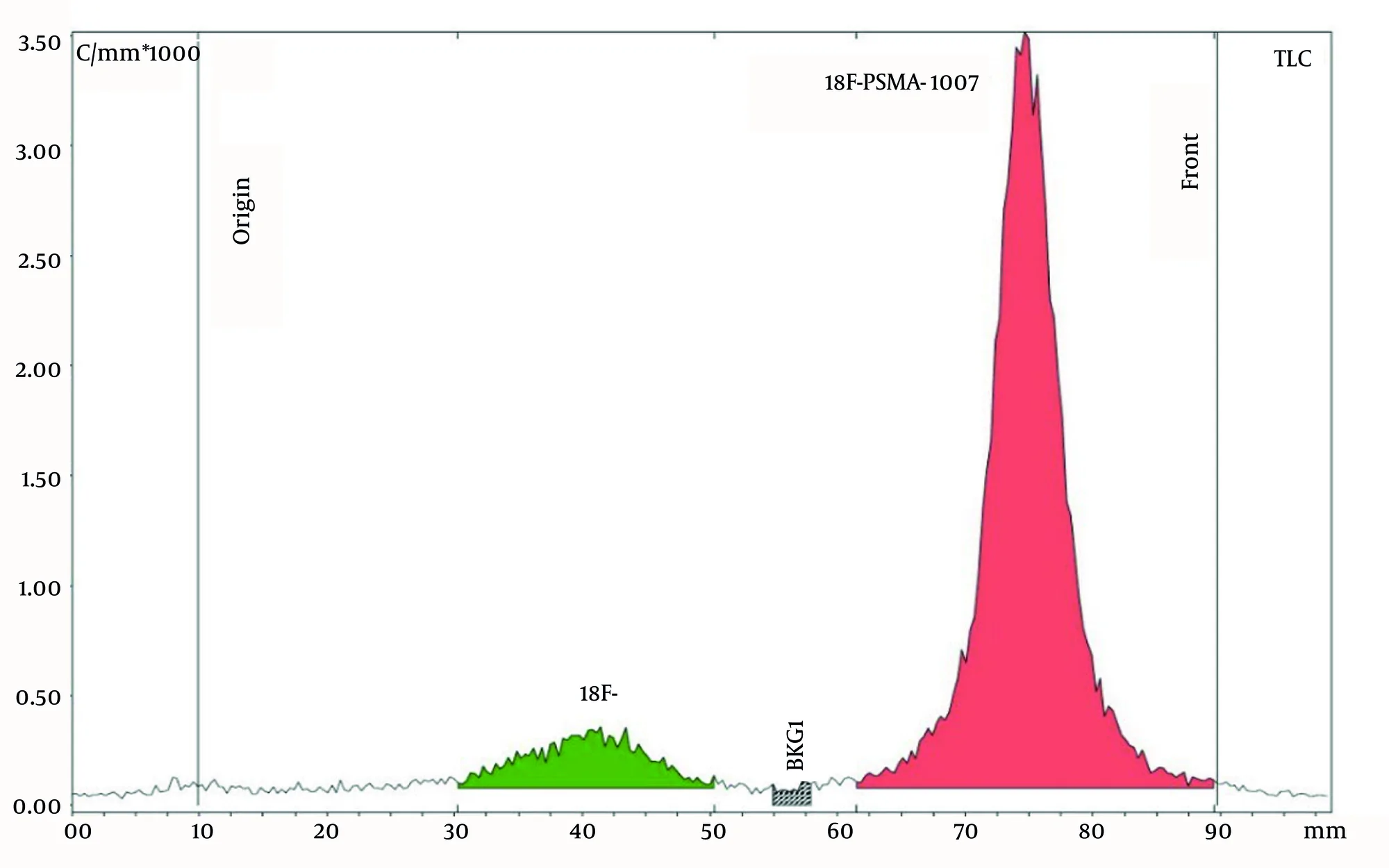
Radio-thin layer chromatography (TLC) chromatogram of 18F-prostate-specific membrane antigen (^18^F-PSMA-1007) containing 9.71 % of free 18F ions

However, approximately 15 hours after the synthesis of 18F-PSMA-1007, the radiochemical purity test was repeated once again to detect potentially free 18F ions. The Radio-TLC instrument showed the presence of free 18F ions in the 18F-PSMA-1007 solution (Figure 7), at an Rf of 0.381. The resulting chromatogram showed that after 15 hours the radiochemical purity of 18F-PSMA-1007 was already 78.8%, and the presence of free 18F ions in the solution was 9.71%.

As previously mentioned, the 18F-PSMA-1007 precursor was dissolved in DMSO. After fluorination, the resulting solution was passed through a C18ec resin column, which retained the target molecule while the remaining solvent was directed to a waste container. The C18ec resin was subsequently washed multiple times with 5 % ethanol. Gas chromatography was used to quantify residual solvents under the conditions listed in Table 1. 

**Table 1.** Gas Chromatographic Conditions for Quantitative Determination of Residual Solvents

Parameters	Values
**Split Ratio**	10
**Injection (inlet) temperature (ºC)**	250
**Detector temperature (ºC)**	300
**Column flow (mL/min)**	3
**Make-up gas (air) flow (mL/min)**	25
**Fuel (hydrogen) flow (mL/min)**	35
**Air flow (for flame only; mL/min)**	250
**Initial (ºC)**	80
**Column oven temperature profile/program (min, ºC)**	(0 - 3, -80); (4 - 6, -150); (6 - 7.5, -250)
**Detector**	FID
**Column**	Length = 30 m; ID = 0.533 mm; layer thickness = 1 mm

**Figure 8.**
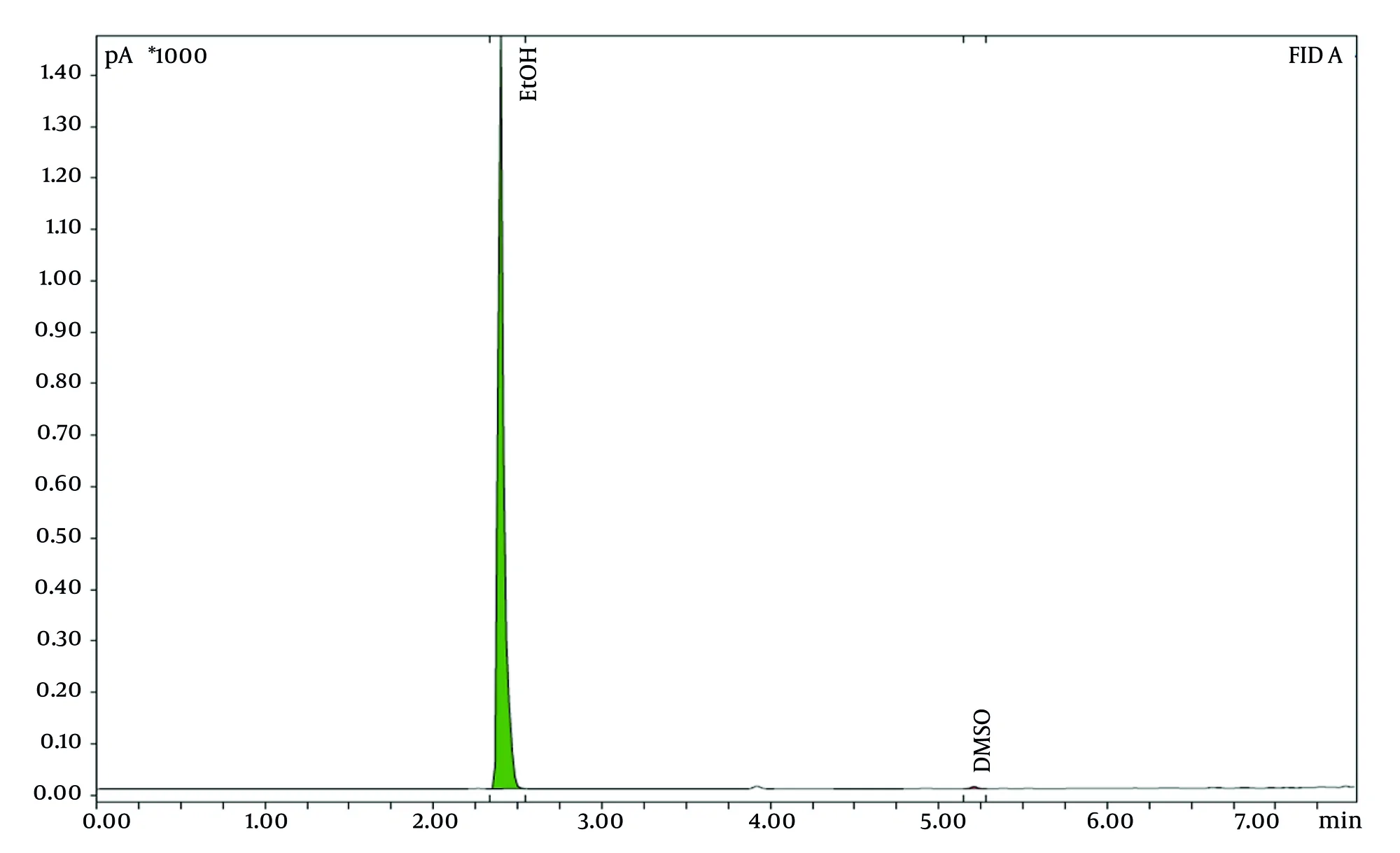
Gas chromatographic analysis for residual solvents in ^18^F-prostate-specific membrane antigen (^18^F-PSMA-1007) solution

Gas chromatographic analysis data (Figure 8) obtained using an Agilent 6850 indicated the presence of DMSO and ethanol in the 18F-PSMA-1007 solution, however, their concentrations are significantly below the maximum permissible values. The following concentrations were calculated: DMSO - 0.025 mg/mL, ethanol - 1.2 %. It should be noted that the presence of ethanol is attributed to the use of a 25 % ethanol solution to elute the target radiopharmaceutical into the dispensing cell at the final stage of synthesis. The low concentration of DMSO in the final solution indicates its near-complete removal from the C18ec resin at the washing stage, confirming the effectiveness of the purification process.

As an intravenous radiopharmaceutical, 18F-PSMA-1007 must be biologically pure before administration to the patient. Therefore, sterility and bacterial endotoxin tests were conducted, respectively, to detect the presence of viable bacteria or fungi as well as gram-negative bacterial lipopolysaccharides (endotoxins). With PTS2005F cartridges (sensitivity of 5 - 0.05 EU/mL) and the PTS Endosafe instrument, the level of bacterial endotoxins was measured after diluting the radiopharmaceutical with Endotoxin-free water (LAL water, Pirotest, Russia). Approximately 15 minutes after the test was started, the PTS Endosafe device reported a bacterial endotoxin level of <1.00 EU/mL, which is within the acceptable limit of 17.5 EU/mL for intravenous solutions. Additional endotoxin testing is summarized in Table 2. 

**Table 2.** Endosafe Charles River Portable Test System Result for ^18^F-Prostate-Specific Membrane Antigen

Sample	Dilution Factor	Reaction Time of 4 Channels (s)	Sample Reaction Time CV (%)	Spike Value (EU/mL)	Spike Reaction Time CV (%)	Spike Recovery (%)	Test Suitability	Sample Value (EU/mL)
^**18**^ **F-PSMA-1007**	20	733	0	0.761	3.2	117	Pass	< 1.00

Abbreviation: ^18^F-PSMA-1007, ^18^F-prostate-specific membrane antigen.

**Figure 9.**
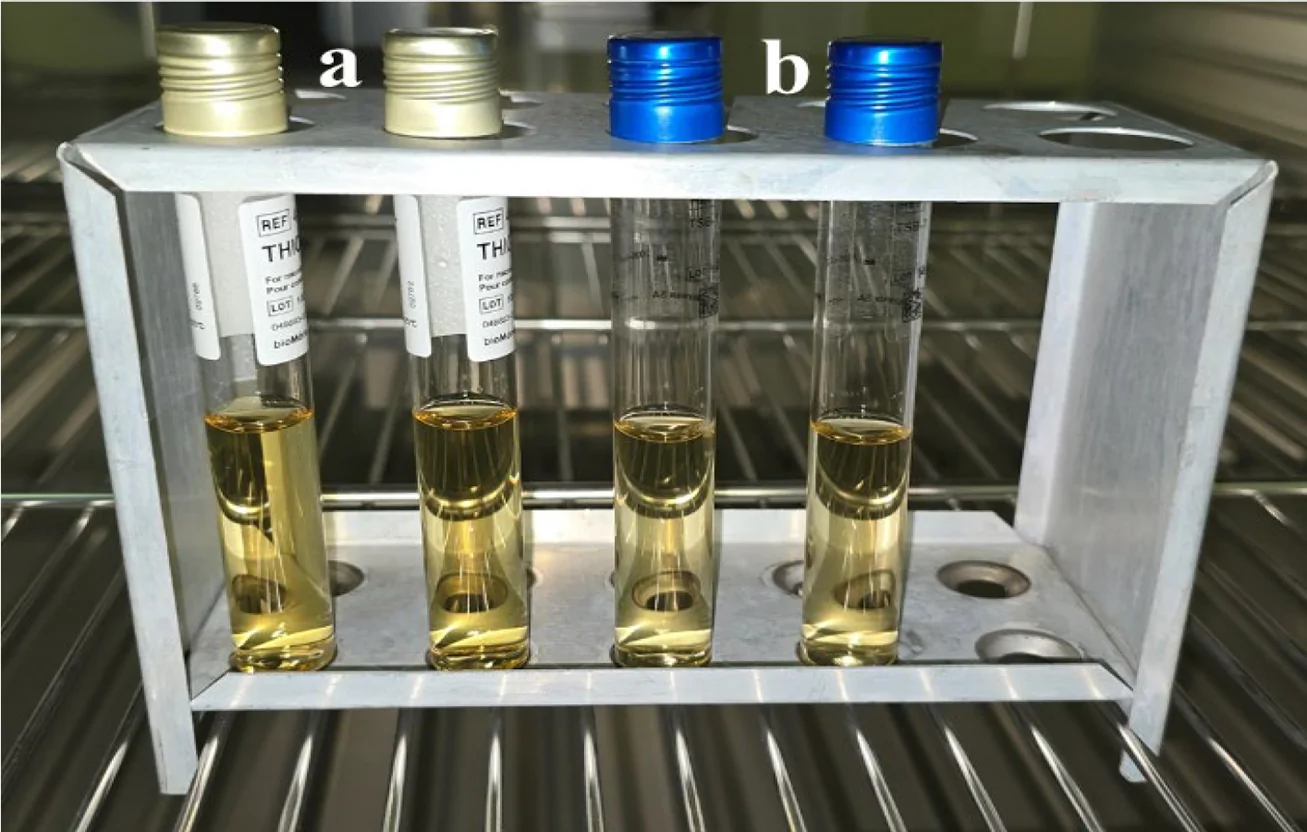
Thioglycollate (A) and Trypticase Soy Broth (TSB) (B) mediums after 14 days’ incubation.

The sterility test was performed only after the radioactivity level had significantly decreased due to its initial radioactive nature. Sterility assessment was performed using 9 mL of Trypticase Soy Broth (TSB) and 9 mL of Clear Thioglycollate Medium growth media for determination of both aerobic and anaerobic bacteria, respectively (40). The samples were incubated in a UFE 600 (Memmert, Germany) incubator at 32±2 °C for 14 days. At the end of the incubation period, no microbial growth was observed (Figure 9). TSB confirmed the absence of microorganisms such as Aspergillus brasiliensis, Bacillus subtilis, and Candida albicans. When using thioglycollate, the preparation was free of Clostridium sporogenes, Pseudomonas aeruginosa, Staphylococcus aureus, and other anaerobic bacteria. These results confirm compliance of the 18F-PSMA-1007 with sterility requirements.

The 18F-PSMA-1007 radiopharmaceutical was found to meet the microbiological purity requirements established for intravenous radiopharmaceuticals based on the outcomes of sterility and bacterial endotoxin testing. According to the stated standards, the analytical data verify that the preparation is safe for additional clinical use and does not pose a risk of microbiological contamination. It should be noted that the obtained results also indicate the preservation of high sterility levels throughout the synthesis process. Since the radiopharmaceutical was dispensed using the Timotheo Kit system (Comecer, Italy), which contains a sterile filter with a pore size of 0.22 μm, the indicators of biological purity additionally confirm the effectiveness of this filling system in ensuring sterile conditions.

Batch release is carried out by a qualified person following the completion of all quality control testing, based on verification that the batch meets predefined quality specifications.

Summarizing all the stages of quality control carried out, it can be confidently stated that the 18F-PSMA-1007 radiopharmaceutical, obtained for the first time in Armenia at the Radioisotope Production Center, fully complies with the relevant requirements of the European Pharmacopoeia and is suitable for PET studies of prostate tumors. Summary indicators of the quality control of 18F-PSMA-1007, along with the corresponding specifications, as well as quality control results for the additional two syntheses, are presented in Table 3. 

**Table 3.** ^18^F-Prostate-Specific Membrane Antigen Radiopharmaceutical Quality Control Data

Parameters	Method	Specification	Result 1	Result 2	Result 3
**Appearance**	Visual	Clear, colorless, or slightly yellow	Colorless solution	Colorless solution	Colorless solution
**pH**	pH measurement	4.5 - 8.5	6.42	6.9	6.7
**TBA (mg/mL)**	TLC	< 0.22	No spot	No spot	No spot
**Radionuclide identification, isotope energy**	γ-spectrometry	γ-photon: 497 - 526 keV; Σ peak: 992 - 1053 keV	520 keV; 1052 keV	504 keV; 1020 keV	518 keV; 1050 keV
**Radionuclide identification, half-life (min)**	Ionization chamber (counter)	105 - 115	107.4	109.5	110.1
**Residual solvents (%, mg/mL)**	Gas chromatography	Ethanol: < 10, DMSO: < 5	1.2, 0.025	0.3, 0.17	5, 0.3
**Radiochemical purity (%)**	Radio-TLC	≥ 91 of total radioactivity (^18^F)	95	96	96
**Bacterial endotoxin (EU/mL)**	LAL test	17.5	< 1.0	< 1.0	< 1.0
**Sterility test**	EP sterility standard test	Sterile (no growth)	Sterile	Sterile	Sterile

Abbreviations: TBA, tetrabutylammonium bicarbonate; TLC, thin layer chromatography; DMSO, dissolved in 1.5 mL of dimethyl sulfoxide; EP, European Pharmacopoeia.

## 5. Discussion

The first synthesis of the radiopharmaceutical 18F-PSMA-1007 and its local application played an important role for the healthcare system of the Republic of Armenia. It opened up new opportunities for ultra-precise studies of prostate cancer in the country.

It should be noted that the use of the above-mentioned production processes for 18F-PSMA-1007 can be more than useful for all those who need to synthesize this radiopharmaceutical on site. In particular, the generation of 18F ions with Cyclone 18Twin, their elution with an aqueous TBA solution, allows for more efficient nucleophilic radiolabeling. In particular, it should be noted that under nitrogen flow conditions, at a temperature of 110°C, the complete removal of water from the TBA solution allows for much higher yield of nucleophilic radiolabeling. This is explained by the fact that 18F ions are much less nucleophilic in an aqueous environment. Therefore, we can note that the temperature and time parameters used in this stage of the reaction are ideal for maximum removal of water molecules.

Further, purification on C18ec resin with 5 % ethanol solution (four times) allows for the maximum removal of possible impurities. As mentioned, the final elution of this radiopharmaceutical to the bottling chamber is carried out with 6 mL of 25 % ethanol solution. An important circumstance is that 2 of the 6 mL are also used for purification of the 18F-PSMA-1007 molecule on the C18ec resin, which also allows for the removal of possible trace impurities. The radiopharmaceutical is sent to the bottling chamber in a volume of 4 mL. Taking into account also the previously added 15 mL of citrate buffer solution, as a result, we have 19 mL of radiopharmaceutical solution, which is more than sufficient for the needs of the center. It should be noted that passing the 18F-PSMA-1007 solution through an SCX cartridge and a sterilizing filter before mixing with the buffer allows for the removal of trace amounts of the TBA molecule and the production of a sterile, patient-safe intravenous radiopharmaceutical.

The results of this study correlate well with previously reported 18F-PSMA-1007 synthesis data using different synthetic platforms. In an experiment using the IBA Synthera 1 platform, decay not corrected radiochemical yields ranged from 35 to 48 %, and radiochemical purities exceeded 90 %, demonstrating high reproducibility of the method (41). In another study (13) performed using the AllInOne 36 module analyzing products with different initial activities, decay not corrected radiochemical yields were approximately 52 % for low and medium activities and 40 % for high activities, with radiochemical purities consistently exceeding 99 %. In this study, 18F-PSMA-1007 was successfully synthesized for the first time in Armenia with decay not corrected radiochemical yields of 52.2 % and a radiochemical purity of 95 %. Two additional synthetic procedures demonstrated consistent reproducibility with yields of 56.69 % and 51.4 %, respectively. Thus, the obtained yield and quality values are comparable with previously known data, which emphasizes the reliability and robustness of the proposed synthesis method.

### 5.1. Conclusions

This work represents the first successful synthesis of the radiopharmaceutical 18F-PSMA-1007 in Armenia, carried out at the Radioisotope Production Center. With a radiochemical yield of 52.2 % and a purity of 95 %, the product passed comprehensive quality control according to the requirements of the European Pharmacopoeia. The following parameters were evaluated: radiochemical and radionuclidic purity, physicochemical characteristics, residual solvent level, sterility, and bacterial endotoxin content. All results demonstrated full compliance with international standards. Of particular importance is the fact that the high quality of the final product was achieved due to the precise organization of all stages of production - from the preparation of consumables to the operation of equipment and conditions inside the production facilities. Two additional syntheses, as well as subsequent quality control results, also demonstrated the repeatability of the process. This confirms the high level of maturity of the technological infrastructure and the professionalism of the personnel.

The launch of local production of 18F-PSMA-1007 has become an important milestone for nuclear medicine in Armenia. Due to this achievement, the first PET investigation with 18F-PSMA-1007 in the country was performed. It not only makes advanced PET diagnostic methods available to patients within the country, but also creates the potential for further development and export focus of the domestic radiopharmaceutical industry. The successfully implemented project serves as a solid foundation for expanding the range of radiopharmaceuticals used in oncology and other medical fields.

## Data Availability

The dataset presented in the study is available on request from the corresponding author during submission or after publication.
